# Whole-Gene Positive Selection, Elevated Synonymous Substitution Rates, Duplication, and Indel Evolution of the Chloroplast *clpP1* Gene

**DOI:** 10.1371/journal.pone.0001386

**Published:** 2008-01-02

**Authors:** Per Erixon, Bengt Oxelman

**Affiliations:** 1 Department of Systematic Botany, Evolutionary Biology Centre, Uppsala University, Uppsala, Sweden; 2 Department of Plant and Environmental Sciences, Göteborg University, Göteborg, Sweden; Ecole Normale Supérieure de Lyon, France

## Abstract

**Background:**

Synonymous DNA substitution rates in the plant chloroplast genome are generally relatively slow and lineage dependent. Non-synonymous rates are usually even slower due to purifying selection acting on the genes. Positive selection is expected to speed up non-synonymous substitution rates, whereas synonymous rates are expected to be unaffected. Until recently, positive selection has seldom been observed in chloroplast genes, and large-scale structural rearrangements leading to gene duplications are hitherto supposed to be rare.

**Methodology/Principle Findings:**

We found high substitution rates in the exons of the plastid *clpP1* gene in *Oenothera* (the Evening Primrose family) and three separate lineages in the tribe *Sileneae* (Caryophyllaceae, the Carnation family). Introns have been lost in some of the lineages, but where present, the intron sequences have substitution rates similar to those found in other introns of their genomes. The elevated substitution rates of *clpP1* are associated with statistically significant whole-gene positive selection in three branches of the phylogeny. In two of the lineages we found multiple copies of the gene. Neighboring genes present in the duplicated fragments do not show signs of elevated substitution rates or positive selection. Although non-synonymous substitutions account for most of the increase in substitution rates, synonymous rates are also markedly elevated in some lineages. Whereas plant *clpP1* genes experiencing negative (purifying) selection are characterized by having very conserved lengths, genes under positive selection often have large insertions of more or less repetitive amino acid sequence motifs.

**Conclusions/Significance:**

We found positive selection of the *clpP1* gene in various plant lineages to correlated with repeated duplication of the *clpP1* gene and surrounding regions, repetitive amino acid sequences, and increase in synonymous substitution rates. The present study sheds light on the controversial issue of whether negative or positive selection is to be expected after gene duplications by providing evidence for the latter alternative. The observed increase in synonymous substitution rates in some of the lineages indicates that the detection of positive selection may be obscured under such circumstances. Future studies are required to explore the functional significance of the large inserted repeated amino acid motifs, as well as the possibility that synonymous substitution rates may be affected by positive selection.

## Introduction

The circular chloroplast genome is in general expected to be a non-recombining unit where large within-genome duplications are rare. Most of its genes are single-copy, occurring in large and small single-copy regions, that are intervened by inverted repeat regions [1]. Substitution rates of chloroplast DNA (cpDNA) are held to be relatively slow and not very variable, although not constant, among lineages [2]–[5]. The gene content is likewise thought to be well conserved [6].

Most reports of positive selection are from the human genome or other model organisms [7], [8], and documented significant positive bias of non-synonymous (dN) over synonymous (dS) substitutions from non-model organisms and/or entire genes are rare (but see [9] and [10]). From an evolutionary perspective it is to be expected that some genes (e.g. those involved in the immune system) can have specific sites that are under positive selection [11], but genes that exhibit positive selection as a whole and with non-synonymous substitutions more or less evenly distributed over the entire length of the gene are clearly more enigmatic and of greater general evolutionary interest.

The chloroplast-encoded *clpP1* (caseinolytic peptidase, ATP-dependent, proteolytic subunit) is part of a gene family encoding *clpP* proteases with six members in *Arabidopsis* of the mustard family Brassicaceae [12]. The other five members are encoded in the nucleus (*clpP2*-*clpP6*) [12]. The main function of the protein is to degrade polypeptides, but the *clpP* proteases are involved in a variety of processes, ranging from developmental changes to stress tolerance [13]. It has been suggested that the *clpP1* gene is essential for plant cell viability [14], [15]. The gene is found in the chloroplast genome of all higher plants and most eukaryotic algae [12]. The structure of the gene and the amino acid composition are highly conserved with 196 amino acid residues distributed over three exons and with two intervening introns, but *Oenothera*, grasses, and the conifer genus *Pinus* lack the two introns. Most green algae also lack introns, but they have roughly the same number of amino acid residues as land plants. However, *Chlamydomonas* and *Scenedesmus* (both Chlorophyceae) have large insertions resulting in a total of 525 and 538 amino acids, respectively. It has been shown that the insertion sequence is not removed during transcription [16].

The present study makes a strong case for the existence of whole-gene positive selection on the chloroplast *clpP1* gene in at least three separate lineages of flowering plants. The positive selection appears to be linked to multiple gene-region duplications, elevation of synonymous substitution rates, and insertion of repetitive amino acid sequence motifs.

## Results

**Figure 1:**
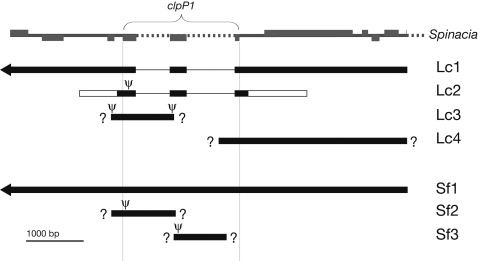
Multiple Copies of the *clpP1* Gene Region Found in Two Species. DNA fragments sequenced from the *clpP1* region (thick black bars) for *Lychnis chalcedonica* (Lc) and *Silene fruticosa* (Sf). Shown in gray is the corresponding region, with the genes (boxes) and introns (dotted lines), in *Spinacia*. Thick white bars indicate non-homologous flanking regions (Lc2 is located differently in the chloroplast genome). Fragments with arrows on the left side consist of a continuous sequence from *rbcL* to *petB* (c. 18 kb). Thin lines mark lack of introns. Ψ indicates stop codon. ? indicates unknown flaking regions.

In all 25 flowering plant species sequenced for this study (21 *Sileneae*, 4 *Oenothera*), there is one copy of the *clpP1* gene located between the *rps12* and *psbB* genes. None of these had frame shifts or premature stop codons, and thus appeared functional. This is in accordance with all published land plant chloroplast genomes. Additional *clpP1* copies were found in two of the species (Fig. 1). Several of the *clpP1* sequences lack introns (Table 1).

**Table 1:** Length variation^a^, PCR primers, and amino acid repeats in the *clpP1* region.

Taxon	*rps12*-*clpP1* spacer	exon 3	intron 2	exon 2	intron 1	exon 1	*clpP1*-*psbB* spacer	PCR primers^b^	Amino acid repeats in exon inserts
*Lychnis chalcedonica* Lc1	113	291	none	292	none	86	506		
*Lychnis chalcedonica* Lc2	55!	231^e^	none	292	none	71	112!	cndhC-F//trnM-R	
*Lychnis chalcedonica* Lc3	125	227^e^	547	>72^e^	?	?	?	rps12-F//clpP/psbH-R8	
*Lychnis chalcedonica* Lc4	?	?	?	?	>216	71	445	clpP/psbH-F2//psbH-R	
*Lychnis abyssinica*	98	234	none	292	none	593	373	rps12-F//clpP/psbH-R5	
*Lychnis flos-cuculi*	102	234	none	292	none	602^d^	380	petL/clpP-F3//clpP-R, rps12-F//clpP/psbH-R5	6×16
*Lychnis flos-jovis*	123	228	597	292	814	71	446	petL/clpP-F3//cp-R7, rps12-F//clpP/psbH-R5	
*Silene fruticosa* Sf1	113	243	620	292	844	659^d^	480		7×8, 7×6, 2×22
*Silene fruticosa* Sf2	122	227^e^	625	>82	?	?	?	clpP-F//clpP/psbH-R8, rps12-F//Sf2-R	
*Silene fruticosa* Sf3	?	?	?	>242^e^	>692	?	?	clpP/psbH-F1//clpPexon1-R	
*Silene conica*	101	372	none	292	none	80	667		
*Silene conoidea*	>26	324	none	292	none	80	>601	rps12-F//clpP/psbH-R6	
*Silene aegyptiaca*	126	228	595	292	851	71	442		
*Silene atocioides*	126	228	603	292	850	71	457		
*Silene pseudoatocion*	124	228	609	292	794	71	446		
*Silene schafta*	126	228	602	292	771	71	445		
*Silene sordida*	126	228	603	292	820	71	443		
*Silene cryptoneura*	131	228	622	292	816	71	443		
*Silene latifolia*	137	228	596	292	837	71	446		
*Silene integripetala*	130	228	597	292	827	71	448		
*Silene uniflora*	128	228	622	292	832	71	445		
*Silene littorea*	136	228	595	292	808	71	446		
*Silene samia*	137	228	586	292	837	71	444		
*Silene sorensenis*	136	228	611	292	800	71	446		
*Silene zawadskii*	136	228	595	292	830	71	448		
*Heliosperma alpestre*	126	228	616	292	829	71	455		
*Oenothera flava*	514	348	587	292	904	71	551	rps12-F//clpP/psbH-R6	
*Oenothera elata*	473	369^d^	none	292	none	89	332	rps12-F//clpP/psbH-R6	2×15
*Oenothera fruticosa*	767	525^d^	none	292	none	71	523	rps12-F//clpP/psbH-R6	2×21
*Oenothera macrocarpa*	519	864^d^	none	292	none	71	455	rps12-F//clpP/psbH-R6	7×16

? no sequence information ! end of insertion.

a All numbers indicate length in base pairs.

b Only indicated if not the complete *rbcL*-*petB* region is sequenced. Primers in bold face match only one of the species paralogues.

c Lc2 is an insertion in the spacer between *ndhC* and *trnV*.

d Exon contains one or several amino acid repeats.

e Exons with stop codon.

**Figure 2:**
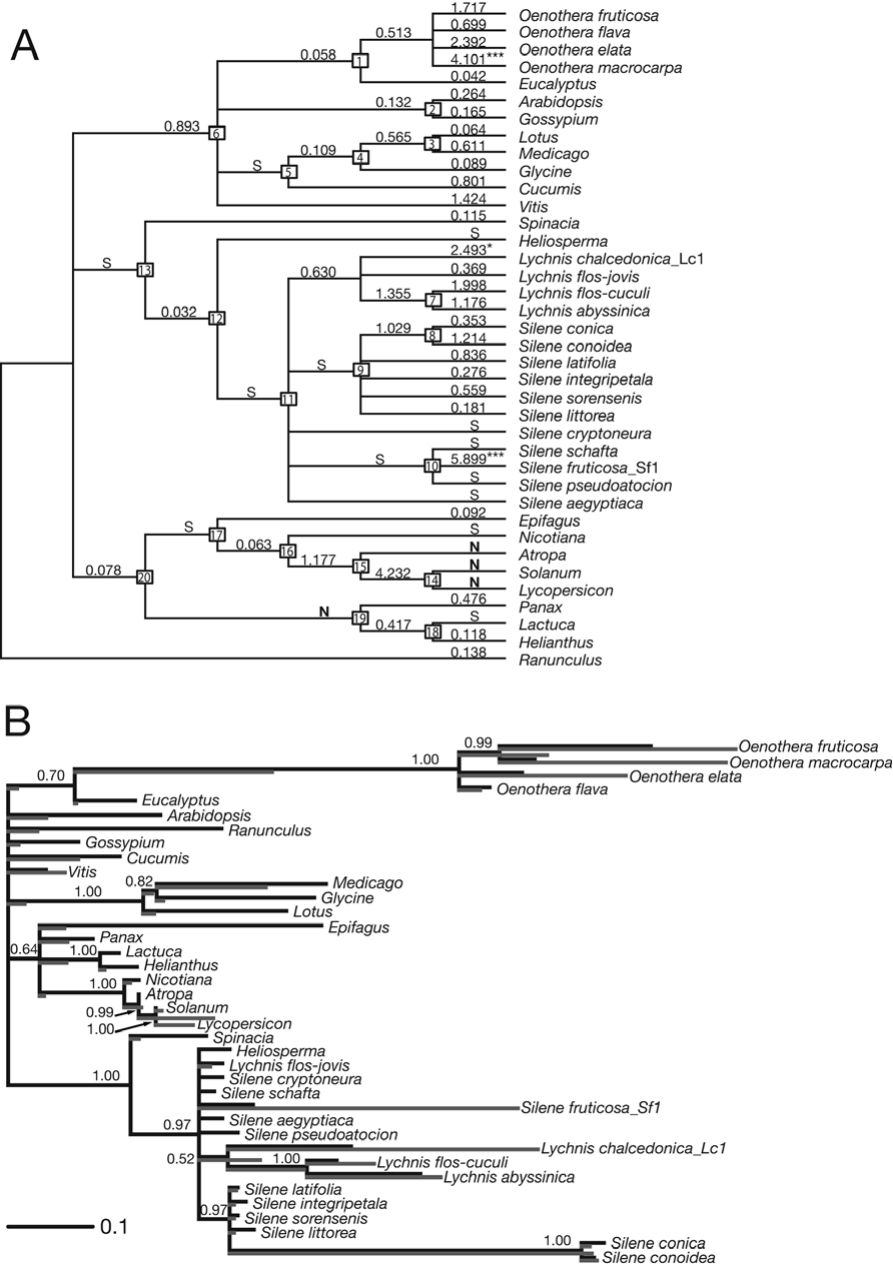
Positive Selection in the Chloroplast *clpP* Gene. A) dN/dS ratios calculated on the Eudicot tree topology. Numbers on nodes indicate classification as follows: 1 Myrtales, 2 Eurosids II, 5 Eurosids I, 6 Rosids, 13 Caryophyllales, 17 Euasterids I, 19 Euasterids II, 20 Asterids [36]; 3 Hologalegina, 4 Fabaceae [37]; 7, 8, 9 subgenus *Behen*, 10 subgenus *Silene*, 11, 12 *Sileneae* [17]; 14 Solaneae, 15 Solanoideae, 16 Solanaceae [38]. Values on branches are the dN/dS ratios, S: dN = 0, N: dS = 0. Ratios significantly above one (Bonferroni-corrected) are indicated by asterisks: ***P<0.001, *P<0.05. B) dN- and dS-branch (in grey and black, respectively) lengths imposed on topology from Bayesian analysis of third codon positions from the *clpP1* exons Numbers on nodes are Bayesian posterior probabilities (Bpp). Only Bpp values>0.50 are shown.

**Figure 3:**
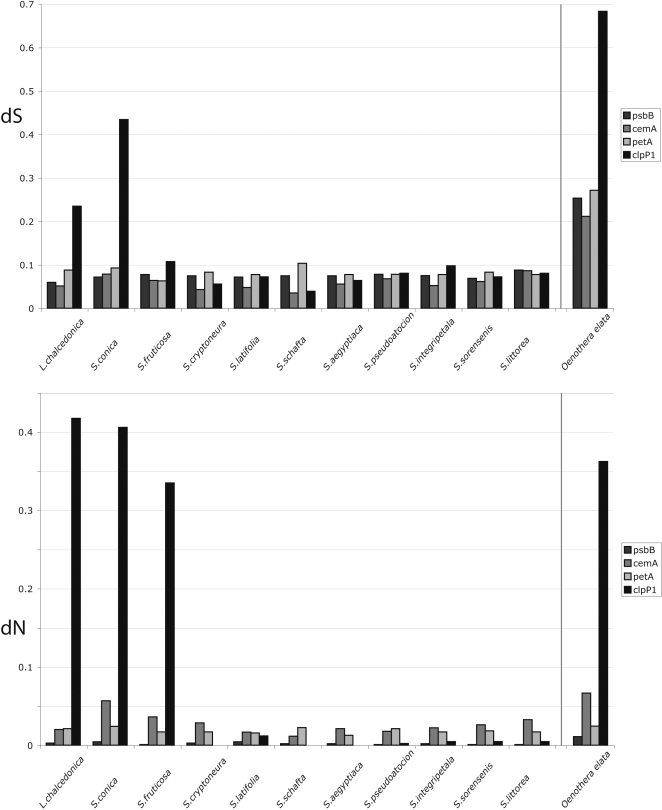
dS and dN Values of Four Chloroplast Genes. Diagram shows the pairwise dS values (top) and dN values (bottom) between eleven species of *Sileneae* and the outgroup (*Heliosperma alpestre*) for four chloroplast genes (*clpP1*; 591 bp, *psbB*; 1527 bp, *cemA*; 639 bp, and *petA*; 963 bp). To the right of the vertical gray line is the pairwise comparison between *Oenothera elata* and *Eucalyptus globulus*, for the same genes.

Six of 21 investigated species of *Sileneae* showed signs of elevated branch lengths in the *clpP1* exons (Fig. 2B). These taxa are distributed in three phylogenetically distinct lineages [17]–[20]: two closely related species in *Silene* subgenus *Behen* (*S. conica* and *S. conoidea*), one species in *Silene* subgenus *Silene* (*S. fruticosa*), and three of four investigated *Lychnis* species (*L. chalcedonica*, *L. flos-cuculi*, and *L. abyssinica*). Three of the six species (*S. conica*, *S. fruticosa*, and *L. chalcedonica*) have been extensively sequenced (>25 kb) also for other, both coding and non-coding chloroplast regions, but do not display such extreme branch lengths elsewhere in the plastid genome [18]. Surprisingly, the synonymous substitution rates in two of these three species are clearly elevated (up to five times) in *clpP1* when compared with other cpDNA genes (Fig. 3). All four investigated species of *Oenothera* had elevated substitution rates. Only *O. flava* possess introns of the four, and it is also the one with the shortest branch length (Fig. 2B). Synonymous substitution rates in the *clpP1* gene of *O. elata* are 2.5–3.2 times higher than other chloroplast genes (Fig. 3).

The position and length of the *clpP1* exons are generally conserved in *Sileneae*, like in most other angiosperms (Table 1), but exceptions were found in the six *Sileneae* species with long branches and the *Oenothera* sequences. *Silene conica* and *S. conoidea* share seven indels, of which the two largest insertions differ in length between the two species. Several of the species had long insertions partially consisting of amino acid repeats in their exons (Table 1).

Analysis of all available seed plant *clpP1* sequences together with the five nuclear encoded gene family members (*clpP2*-*clpP6*) from *Arabidopsis* and *Oryza* strongly indicated (posterior probability 1.00) that the divergent sequences found in this study are of chloroplast origin (Fig. S1). The relationships within *Sileneae* in the gene family analysis, where all the long branches group together, are at odds with all previous cpDNA analyses (e.g. [18]). The analysis of only third positions from the *clpP1* exons did, however, result in a topology compatible with other analyses of sequences from other cpDNA regions (Fig. 2B). For example, despite its very long branch, the *Silene conica* and *S. conoidea* clade grouped together with other members of *Silene* subgenus *Behen* (Fig. 2B).

**Figure 4:**
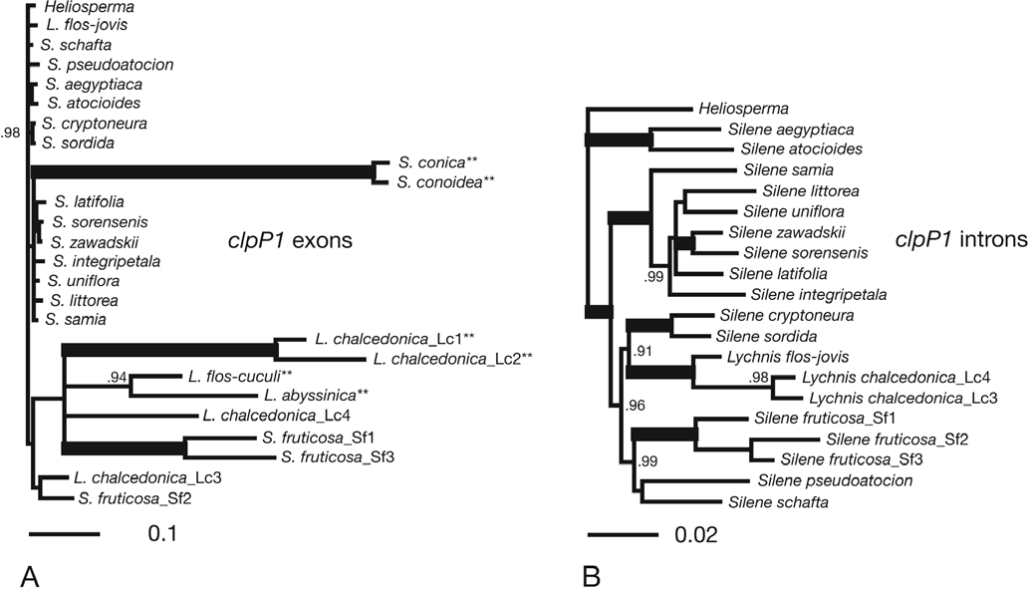
Bayesian Consensus Phylograms of the Tribe *Sileneae.* A) The *clpP1* exon sequences, and B) the *clpP1* intron sequences. Branches in bold have Bayesian posterior probabilities (Bpp) of 1.00. Only Bpp values above 0.90 are shown at nodes. Note that A and B are drawn at different scales. ** indicates taxa that lack introns in *clpP1*.

The *Sileneae* phylogenies based on *clpP1* exon (Fig. 4A) and intron (Fig. 4B) sequences were substantially different. Intron sequences from *Lychnis chalcedonica* and *Silene fruticosa* did not show signs of elevated substitution rates relative to other cpDNA regions, and the phylogeny (Fig. 4B) is compatible with other *Sileneae* phylogenies based on cpDNA [18].

We used the method of Yang [21] to investigate the ratio of non-synonymous to synonymous substitutions (dN/dS, ω) and found that this ratio varies significantly among lineages (P<0.0001), both under a generally accepted eudicot phylogeny (Fig. 2A) and under the tree topology from Bayesian analysis of only third positions from the *clpP1* exons (Fig. 2B). Several branches in both *Sileneae* and *Oenothera* resulted in ω>1 (Fig. 2). In addition, the branches in Solanaceae have high ω-values, despite shorter branch lengths. Three branches have ω that are significantly higher than one after Bonferroni correction (table 2, Fig. 2A). The second topology (Fig. 2B) gives very similar ω-values, but the values of the three branches with significant positive selection are higher still, and two internal branches not resolved in the first topology (Fig. 2A) receive ω-values>2.0 (Fig. 2B).

**Table 2:** Branches with dN>dS in the phylogeny in figure 1A.

Branch leading to:	dN/dS	-lnL	χ^2^ = Δ2lnL	P_uncorrected_	P_Bonferroni_
*O. fruticosa*	1.717	7019.4213	4.0108	0.0452 *	n.s.
*O. elata*	2.392	7020.8512	6.8706	0.0087 **	n.s.
*O. macrocarpa*	4.101	7027.0673	19.3028	0.00001 ***	0.00067 ***
*Vitis*	1.424	7018.3996	1.9674	0.161	n.s.
*L. chalcedonica*	2.493	7023.2954	11.759	0.0006 ***	0.036 *
*L. flos-cuculi*	1.998	7018.7147	2.5976	0.107	n.s.
*L. abyssinica*	1.176	7017.5145	0.1972	0.657	n.s.
node 7	1.355	7017.6766	0.5214	0.470	n.s.
*S. conoidea*	1.214	7017.4239	0.016	0.8993	n.s
node 8	1.029	7018.1340	1.4362	0.231	n.s.
*S. fruticosa*	5.899	7029.2347	23.6376	0.000001 ***	0.00007***
*Atropa*	N^a^	7018.5259	2.22	0.136	n.s.
*Solanum*	N^a^	7018.0874	1.343	0.247	n.s.
*Lycopersicon*	N^a^	7021.3030	7.7742	0.0052 **	n.s.
node 14	4.232	7020.2991	5.7664	0.016 *	n.s.
node 15	1.177	7018.1436	1.4554	0.228	n.s.
node 19	N^a^	7017.4737	0.1156	0.734	n.s.

a Branches with only non-synonymous substitutions.

We reject the idea that the observed open reading frames in the *clpP1* sequences of *Silene conica* and *S. conoidea* have persisted by chance alone under a neutral model of evolution, because only 0.69% of simulated sequences with a branch length of 0.25 from the *S. latifolia* sequence appear to have remained functional (98.66% had premature stop-codons and an additional 0.65% lacked a proper start-codon). The average number of premature stop codons per simulated sequence was 4.3.

## Discussion

Our results indicate highly elevated substitution rates in the chloroplast *clpP1* exons in *Sileneae* and in *Oenothera*. Although long branch lengths also could indicate high ages, we argue that this is unlikely because the comparisons with other genes (Fig. 3) which, unless acquired by horizontal transfer, must be of the same age. Even if the topological relationships sometimes are ambiguous, any resolution of the trees in Fig. 2 will show that the sister group has a significantly shorter branch. As sister groups by definition has the same age, either the short branch has undergone a substitution rate decrease or vice versa. This supports the explanation for the long *clpP1* branches as likely to have been the result of a substitution rate increase.

In *Sileneae*, there are at least three independent increases in substitution rates. Loss of introns in *clpP1* appears correlated with elevated substitution rates in several lineages. In two of the *Sileneae* lineages, high substitution rates are accompanied with both gene duplications and significant positive selection.

Even if the rate of synonymous substitutions in the *clpP1* gene for the *Sileneae* species with generally elevated substitution rates appears higher than that of other chloroplast genes for the same taxa (Fig. 3), most of the total substitution rate increase can be ascribed to non-synonymous substitutions. A generally elevated substitution rate for a whole genome such as the mitochondrial genome of some species of *Plantago* could be explained by, e.g. an increased amount of oxygen free-radicals [22], but elevated substitution rates in, and exclusive to, a specific gene are harder to explain. The very long branch leading to *Silene conica* and *S. conoidea* is puzzling in this context, because the rates of both synonymous and non-synonymous substitutions are very high, and there is no significant positive selection. The pattern is similar, but less extreme, in *L. chalcedonica* (Fig. 3). The length of the branches leading to *Solanum*/*Lycopersicon* (Solanaceae), within the Fabaceae, to *Vitis*, and to *Cucumis* might also indicate elevated substitution rates, but this is much less striking than in the long *Sileneae* branches and in *Oenothera* (Fig. S1).

By comparing synonymous and non-synonymous substitutions, we were able to detect statistically significant positive selection at the gene level on three branches. However, we anticipate that the actual duration of the episodes of positive selection in the tree might be larger. The power of the tests employed here is relatively low, i.e. positive selection is difficult to detect even if it exists at many sites [7]. Recently, methods have been developed to detect positive selection on individual codons for specific branches (e.g. the branch-site likelihood method [23]). These methods are generally more powerful and their utilization has resulted in a marked increase in the number of published reports of positive selection [24]. However, simulations by Zhang [24] showed that the power of these methods comes at a cost in the form of high levels of false positives.

Under the assumption that substitutions in non-coding sequences are selection-wise neutral, elevated substitution rates in exon sequences compared to introns provide an indication of positive selection [25]. By comparing the branch lengths of the exon tree (Fig. 4A) and the intron tree (Fig. 4B) it is apparent that this is the case in *S. fruticosa* and probably also in *Lychnis chalcedonica.* For example, the uncorrected pairwise base distance between *S. fruticosa* (Sf1) and *S. schafta* is 0.23 in exons, but 0.05 in the introns, and for *L. chalcedonica* (exon: Lc1, intron: Lc3/Lc4 combined) and *L. flos-jovis* these figures are 0.30 and 0.04, respectively. Finally, *Oenothera flava* (the only *Oenothera* species in this study to have introns) shows more variability in exons than in introns compared to *Eucalyptus* (0.26 and 0.18, respectively), although the difference is less striking for this taxon.

The *clpP1* exon sequences that show the most extreme substitution rates are those of *Silene conica* and *S. conoidea*, but because they lack introns in the gene, the exon/intron comparison cannot be made. The variability in the gene is approximately an order of magnitude higher (synonymous and non-synonymous substitutions contributing roughly equally to the increase) than that of spacer-regions in the cpDNA of *S. conica* (see below). The pairwise base difference between *Silene conica* and *Silene latifolia* in intergenic spacers is on average 0.03 (*rbcL*/*accD*: 0.037, *petA*/*psbJ*: 0.035, *psbE*/*petL*: 0.028, *rpl20*/*rps12*: 0.022, data from [18]), but the difference in the *clpP1* gene is 0.31. Despite the very divergent sequences, the dN/dS ratios did not indicate positive selection acting within this group. Because ratios around 1 implicate absence of both positive and purifying selection, this indicates that the *S. conica* and *S. conoidea* sequences may have lost their function. However, the simulation experiment strongly rejected the hypothesis that the absence of stop codons can be explained by chance alone. Further support for this is given by the fact that *Silene conica* and *S. conoidea* have seven indels in the *clpP1* sequence, all of lengths that are multiples of three. The existence of these indels that do not distort the reading frame is in itself strong evidence for maintained gene function. Finally, even if *S. conica* appears to have a somewhat elevated cpDNA substitution rate in general [18], it seems unlikely that the very high substitution rates in *clpP1* would be the effect of lost function. Xing and Lee [26] found that alternative splicing could greatly relax selection pressure (measured as dN/dS). This effect was accompanied by a strong decrease in synonymous substitutions. Because we observe a strong increase in synonymous substitutions in our data (Fig. 3), this explanation too, seems unlikely in this particular case.

Whether there is a causal relationship between the increase in synonymous and non-synonymous rates in *Silene conica/conoidea* remains unclear. However, there are other indications that positive selection of *clpP1* is correlated with increased synonymous substitution rates; *Lychnis chalcedonica* and *Oenothera elata* also have elevated synonymous substitution rates (Fig. 3). Some of the other branches in the eudicot *clpP1* tree (Fig. 2) have combinations of branch lengths and dN/dS ratios that indicate a more widespread occurrence of positive selection of the gene. In particular the branch leading to *Solanum*/*Lycopersicon* (node 14 in Fig. 2A) that has a dN/dS ratio significantly higher than 1 before Bonferroni-correction (Table 2), but also the branches leading to node 6 (Fig. 2A), *Medicago*, *Vitis*, and *Cucumis* seem interesting targets for further investigations.

In a systematic search for positive selection in higher plants based on almost 140,000 embryophyte gene sequences from GenBank, very few cases of ω values above one were found when averaging over whole genes [27]. Only in two cases were ω>2, and both of these were sequence pairs of nuclear encoded genes [27]. This illustrates how unusual our findings are. The recent report on positive selection in the chloroplast gene *rbcL* [10] clearly shows that specific sites in that gene are under positive selection in a wide range of land plants. Our study gives indications that positive selection in the *clpP1* gene might be widespread in flowering plants. In *rbcL* it is only a small proportion of the sites that appear to be under positive selection [10], whereas in *clpP1* a very large proportion of the sites are affected.

In the present study, the rates of synonymous substitutions are rather conserved with respect to different taxa and genes, with the important exception of the species undergoing rapid evolution in the *clpP1* gene (Fig. 3). None of the “normal” taxa or genes shows as high dS rates as the *clpP1* gene from those three species. The degree of elevated dS rates also indicates an interesting pattern: the species with the strongest estimated positive selection has the least elevated dS rate and vice versa, i.e. the rate of non-synonymous substitutions are roughly the same among the three species.

Elevated evolutionary rates due to positive selection or relaxed selective constraints are often preceded by gene duplication [28]. We detected extra *clpP1* gene copies only in *Lychnis chalcedonica* and *Silene fruticosa*. Indeed, the completely sequenced chloroplast genome of *Oenothera elata* (NC_002693) contains only one copy of *clpP1*.

Only one of the four *clpP1* copies in *L. chalcedonica* is potentially functional (Lc1), i.e. the others contain stop codons or are incomplete. The intron-containing Lc4 fragment shows obvious signs of elevated substitution rates in *clpP1*, although less so than Lc1. The Lc3 copy, apparently a pseudogene, is less divergent than the other copies in *Lychnis chalcedonica*. This does not seem to be an artifact due to missing data, because in the region where sequence information for Lc1, Lc2, and Lc3 overlap the uncorrected distance between Lc1/Lc2 and *L. flos-jovis* (the “normal” *Lychnis* species in this study) is 30.3%/34.6%, whereas between Lc3 and *L. flos-jovis* it is 3.9%. Thus, in absence of a formal phylogenetic analysis of the *clpP1* copies in *L. chalcedonica*, we may speculate that at least the duplication leading to Lc3 preceded the onset of positive selection. In *S. fruticosa*, Sf2 is markedly less divergent than the two other copies, and thus also probably the result of an ancient duplication preceding the non-synonymous rates increase. In the region where sequence information for Sf1 and Sf2 overlaps the uncorrected distance between Sf1 and *S. schafta* is 22.6%, whereas between Sf2 and *S. schafta* it is 5.2%. Both these cases may thus agree with one of few documented cases where gene duplication precedes the onset of positive selection [29]. It may be that positive selection, under some circumstances, can be triggered by duplication rather than being an expected outcome of it.

The very large insertions (174 to 197 amino acids) found in the *clpP1* exon 1 of *Silene fruticosa* (Sf1), *Lychnis flos-cuculi*, and *L. abyssinica* do not cause frame shifts or stop codons. To our knowledge, the effect of indel evolution has not been studied in relation to positive selection. It is possible that repetitive insertions are beneficial, because given that the repeats are in multiples of three nucleotides, they reduce the probability of stop codons, while possibly fostering new phenotypic variants.

### -Conclusions

In our study, four distantly related taxa or groups of taxa (*Oenothera*, *Silene fruticosa*, *Silene conica/conoidea*, and *Lychnis chalcedonica/flos-cuculi/abyssinica*) exhibit substitution rates in *clpP1* exon sequences that are hitherto unprecedented in the chloroplast genome. We conclude that these high evolutionary rates are correlated with positive selection of *clpP1* in the evolutionary histories of at least three of these four groups. In the case of *Lychnis*, this was probably preceded by a duplication of a segment including *clpP1*, *psbB*, *psbT*, *psbN*, and *psbH*, but only the *clpP1* gene shows signs of positive selection. We cannot rule out the possibility of gene duplications as a causal agent in the other three cases, because duplicates may either be ambiguous, extinct, or undetected. One of the major aspects of the present results is that they indicate that positive selection may be accompanied by elevated synonymous substitution rates in some cases. If this indeed proves to be the case it will have far-reaching consequences for our ability to detect positive selection. Also, the fact that positive selection appears to have originated in at least three closely related lineages of *Sileneae* calls for caution when interpreting plastid data at population and phylogenetic levels (cf. [9]). The relationship between cpDNA duplications, increased substitution rates, positive selection, and indel evolution in the chloroplast genome needs further scrutiny, and plant *clpP1* appears to constitute an excellent model system for such studies.

## Materials and Methods

### -Taxon sampling

All seed plant *clpP1* sequences on GenBank [30] as of May 15th 2006, as well as the nuclear *clpP2-6* from *Oryza* and *Arabidopsis*, were downloaded; in addition, 21 species of *Sileneae* and four species of *Oenothera* were sequenced for the gene (Table S1). The sampling of *Sileneae* species follows that of Erixon and Oxelman [18], but *Silene conoidea* and *Lychnis abyssinica* were added after the observation that closely related taxa exhibited extremely high substitution rates. The inclusion of *Oenothera* in the study was deemed important because a survey of complete chloroplast genomes on GenBank revealed that *Oenothera elata* (NC_002693) lacks introns and shows signs of elevated substitution rates. The four species of *Oenothera* were chosen to represent different sections in the genus [31].

### -DNA Amplification and Sequencing

All 21 *Sileneae* species (except *Silene conoidea*, *Lychnis flos-cuculi*, *Lychnis flos-jovis*, and *Lychnis abyssinica*) were amplified and sequenced in a continuous region from the end of *rbcL* to the beginning of *petB* corresponding to c. 18 kb in *Spinacia*. DNA preparation, amplification, sequencing, and general primers follow Erixon and Oxelman [18].

**Figure 5:**
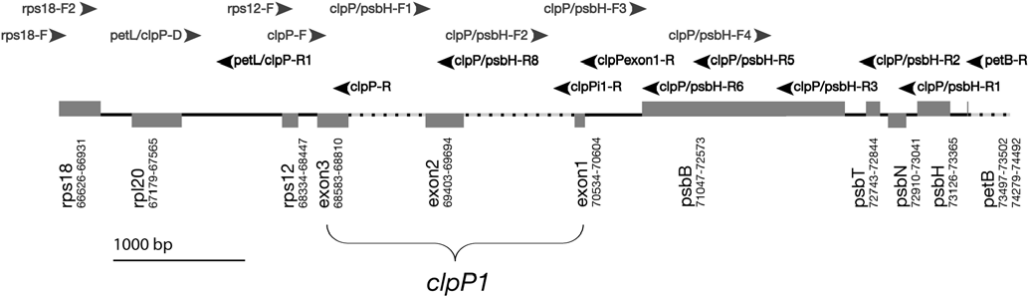
The *clpP1* Gene and Flanking Regions with Primer Sites. Approximate position of general primers for *Sileneae* [18] with reference to *Spinacia*. Boxes correspond to protein coding genes with their name and position in *Spinacia*. Boxes above the line denote genes that are transcribed from left to right, and those below are transcribed from right to left. Dotted lines represent introns.

The *clpP1* region in *Oenothera* was amplified with the PCR primers rps12-F and clpP/psbH-R6 (Fig. 5). For sequencing of this product, eight primers were constructed (Table S2). In addition, some specific primers were made for *Silene fruticosa* and *Lychnis chalcedonica*, either to amplify a specific sequence copy or to sequence through large insertions (Table S2).

### -Alignment

The amino acid sequences from *Arabidopsis* and *Oryza* for all five nuclear members of the *clpP* gene family were aligned using ClustalW version 1.83 [32], with default settings, together with chloroplast *clpP1* amino acid sequences. The nuclear sequences corresponding to the 21 first amino acids in the chloroplast *clpP1* gene of *Spinacia* were excluded prior the analysis, because the alignment responded strongly even to small changes in the parameters. All sequences corresponding to the six last amino acids of *Spinacia clpP1* were also excluded, due to extreme length variation both within *clpP1* and among the other gene family members. The amino acids were only used in the alignment process; all analyses were done on nucleotide sequences.

All non-*clpP1* exon DNA sequences were manually aligned, using the principles of Oxelman et al. [33], in the sequence alignment editor Se-Al version 2.0a11 (http://evolve.zoo.ox.ac.uk/).

### -Phylogenetic analysis

Bayesian phylogenetic analyses were performed with MrBayes version 3.1.2 [34]. For both the gene family data set (72 terminals) and a restricted eudicot *clpP1* data set (38 terminals, see below), substitutions were modeled with the GTR+Γ model, which received highest AIC scores according to MrAIC.pl version 1.4 (http://www.abc.se/∼nylander/) together with PHYML version 2.4.4 [35]. The large data set ran for 50 million generations with six MCMC chains (temperature (t) = 0.2) and four independent runs with trees sampled every 1000th generation. The eudicot data set ran for 20 million generations with four MCMC chains (t = 0.2) and two independent runs with trees sampled every 100th generation. The first 50% of the sampled trees were, in both analyses, discarded as burn-in.

Bayesian analyses, with the same settings as above except that number of generations were 10 million, were performed on: a) a matrix of only third positions from the Eudicot data set, b) a matrix of *Sileneae clpP1* intron sequences, and c) a matrix of *Sileneae clpP1* exon sequences.

### -Detection of positive selection

PAML version 3.14 [21] was used to calculate the non-synonymous (dN) and synonymous (dS) substitutions rates, and the ratio (ω) between them. To test for variation in ω among the branches, the likelihood for the data under a model with fixed ω (estimated from data) for the entire tree (m0) was compared to a model allowing for the branches to have different ω (m1). To test the null hypothesis that addition of a ω parameter for each branch does not increase likelihood of the data, a likelihood-ratio test was performed, where the test parameter is assumed to follow the chi-square distribution with the degrees of freedom equal the number of tree branches −1. The null hypothesis of absence of selection on individual branches was tested by comparing the maximum likelihoods from the m1 analysis to maximum likelihoods for models with free ω for all branches except that the ω of the branch under consideration was set to 1 (m2). This test has one degree of freedom. Only branches with ω>1.0 were tested, but because these were detected *a posteriori*, the probabilities were Bonferroni corrected [7].

### -Topologies used for estimates of dN/dS

Two different tree topologies were used for calculations of dN and dS values. The first topology (Fig. 2A) was based on the classification of the Angiosperm Phylogeny Group II [36] at the family level and on strongly supported within-family relationships published elsewhere [17]–[20], [37], [38] . The sistergroup relationship between *Silene conica* and *S. conoidea* has not been published before, but is based on their very similar morphology, i.e. they belong to the section *Conoimorpha* Otth, which is characterized by a calyx morphology and a basic chromosome number, both of which are unique in *Sileneae*. Five *Silene* sequences were excluded from this analysis; these were identical or almost identical (≤4 bases different) to at least one of the non-excluded sequences. Only sequences with the entire *clpP1* coding region were included in the analyses detecting for positive selection.

To explore the effect of the tree topology on the outcome a second substitution rate analysis was performed using the 50% majority-rule consensus phylogeny from the Bayesian analysis of third positions (Fig. 2B). This topology is incompatible with the first topology with respect to the relations within Fabaceae. Another substantial difference is in the level of resolution. Eight more branches are unresolved in the second phylogeny compared to the first and two relationships are resolved only in the second (in *Oenothera* and in *Lychnis*, see Fig. 2).

### -Synonymous substitution rate comparison

In order to obtain an estimate of the synonymous substitution rates in the *clpP1* exons in *Sileneae* we made pairwise comparisons between eleven *Silene*/*Lychnis* species and *Heliosperma* (outgroup). These estimates were compared with data from three other chloroplast genes (*psbB*, 1527 bp; *cemA*, 639 bp; *petA*, 963 bp). The reason for choosing these particular genes was that they are the only large genes present in the 18 kb fragment sequenced for all the twelve taxa [18], and when Goremykin et al [39] compared the synonymous substitution rates for these genes of ten angiosperms relative to *Pinus*, the average dS values were similar and *clpP1* had the lowest value (*clpP1*, 0.86; *cemA*, 0.89; *psbB*, 1.00; *petA*, 1.53). We also made a pairwise comparison of dS values for the same genes from the complete chloroplast genomes of *Oenothera elata* and *Eucalyptus globulus*. PAML version 3.14 [21] was used for all estimates.

### -Indirect test for loss of function

In the absence of expression studies, we conducted a simple test to evaluate if the observed open reading frames of sequences with highly elevated rates could have persisted by chance. Ten thousand sequences were generated with SeqGen version 1.3.2 [40] under the JC69 model of evolution using the *Silene latifolia* sequence as ancestral and a branch length of 0.25. The branch length was chosen to be considerably smaller than the longest branch observed in the substitution rate analysis, namely the one leading to *S. conica*/*S. conoidea*, i.e. 0.47 (Figures 2A). A simple substitution model was chosen to simulate neutral evolution. Since the chloroplast DNA sequences have an AT-bias, the JC69 model will result in a conservative test, because an excess of A and T will increase the number of simulated stop codons simply because these are AT-rich (TAA, TAG, and TGA).

## Supporting Information

Figure S1 *clpP* gene family phylogeny. A) Bayesian consensus phylogram of all seed plant *clpP1* sequences and the nuclear *clpP2-clpP6* for *Arabidopsis* and *Oryza*, and B) without *Oenothera* and the long *Sileneae* branches. Numbers on nodes are Bayesian posterior probabilities (Bpp). Branches in bold have Bpp = 1.00. Only Bpp>0.90 are shown. Each * indicates a missing intron. Y indicates pseudogene. ? indicates incomplete gene (only exon 1) without stop codon.
(8.35 MB TIF)

Table S1 Plant taxa, vouchers for the sequences obtained in this study, and GenBank accession numbers
(0.04 MB DOC)

Table S2 Specific primers
(0.02 MB DOC)
