# Supplementary figures and images for: Whole-Gene Positive Selection, Elevated Synonymous Substitution Rates, Duplication, and Indel Evolution of the Chloroplast clpP1 Gene
Source: PLoS One. 2008 Jan 2;3(1):e1386. doi: 10.1371/journal.pone.0001386 (PMC2148103; doi:10.1371/journal.pone.0001386)

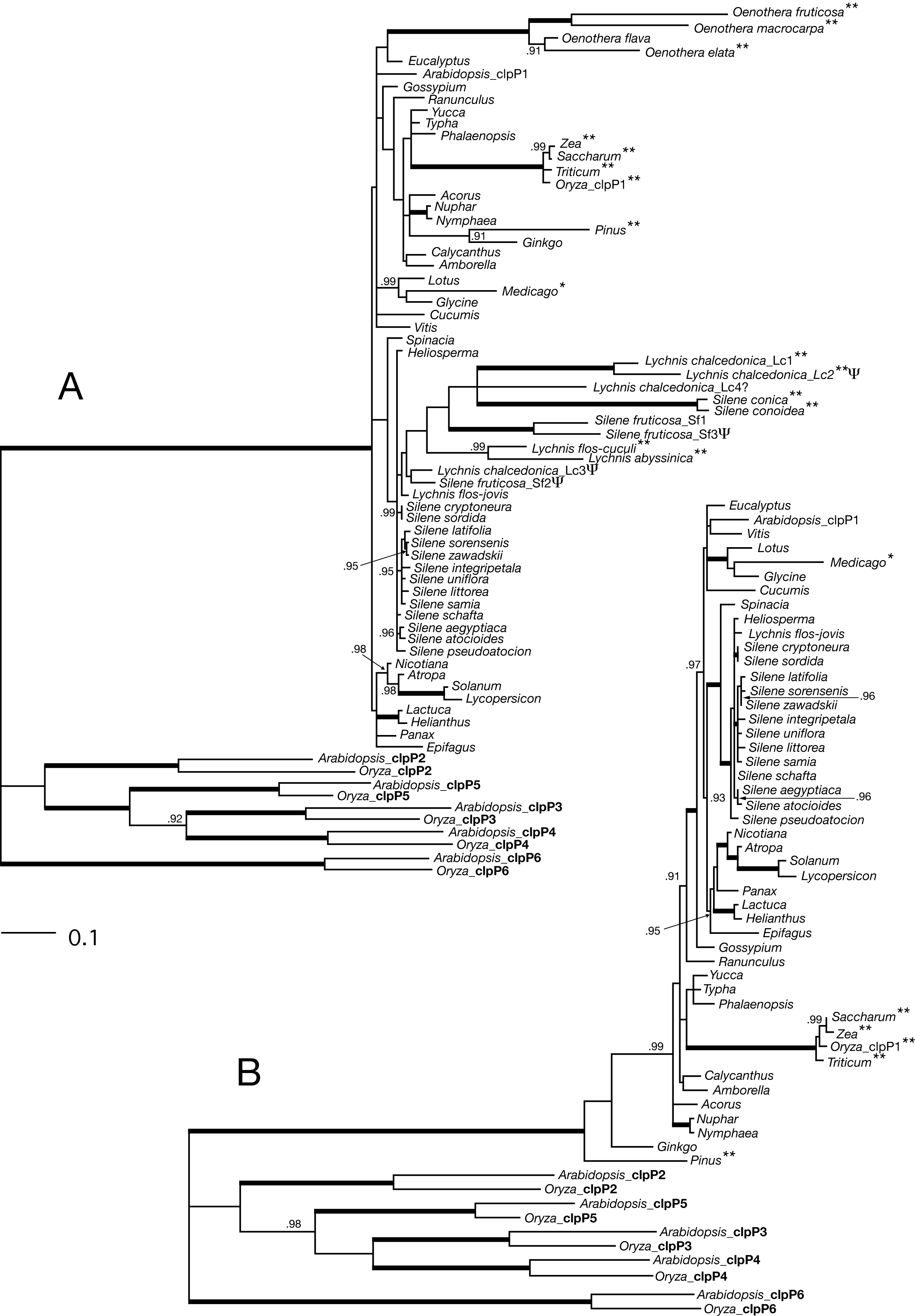

Supplement: Figure S1 — clpP gene family phylogeny. A) Bayesian consensus phylogram of all seed plant clpP1 sequences and the nuclear clpP2-clpP6 for Arabidopsis and Oryza, and B) without Oenothera and the long Sileneae branches. Numbers on nodes are Bayesian posterior probabilities (Bpp). Branches in bold have Bpp = 1.00. Only Bpp>0.90 are shown. Each * indicates a missing intron. Y indicates pseudogene. ? indicates incomplete gene (only exon 1) without stop codon. (8.35 MB TIF) [file pone.0001386.s001.tif]
