# Supplementary material for: Whole-Gene Positive Selection, Elevated Synonymous Substitution Rates, Duplication, and Indel Evolution of the Chloroplast clpP1 Gene
Source: PLoS One. 2008 Jan 2;3(1):e1386. doi: 10.1371/journal.pone.0001386 (PMC2148103; doi:10.1371/journal.pone.0001386)
Supplement: Table S1 — Plant taxa, vouchers for the sequences obtained in this study, and GenBank accession numbers (0.04 MB DOC) [file pone.0001386.s002.doc]

Table S1. Plant taxa, vouchers for the sequences obtained in this study, and GenBank accession numbers

| Species and sequence | Voucher | GenBank acc.no. |
| --- | --- | --- |
| *Acorus calamus* (Schott) Engl. |  | AJ879453 |
| *Amborella trichopoda* Baillon |  | NC_005086 |
| *Arabidopsis thaliana* (L.) Heynh. clpP1 |  | NC_000932 |
| *Arabidopsis thaliana* (L.) Heynh. clpP2 |  | NM_122220 |
| *Arabidopsis thaliana* (L.) Heynh. clpP3 |  | AF370528 |
| *Arabidopsis thaliana* (L.) Heynh. clpP4 |  | AY042832 |
| *Arabidopsis thaliana* (L.) Heynh. clpP5 |  | AY084394 |
| *Arabidopsis thaliana* (L.) Heynh. clpP6 |  | NM_101047 |
| *Atropa belladonna* L. |  | NC_004561 |
| *Calycanthus floridus* L. |  | NC_004993 |
| *Cucumis sativus* L. |  | NC_007144 |
| *Epifagus virginiana* (L.) W.Bart. |  | NC_001568 |
| *Eucalyptus globulus* Labill. |  | AY780259 |
| *Ginkgo biloba* L. |  | DQ069356 |
| *Glycine max* (L.) Merr. |  | NC_007942 |
| *Gossypium hirsutum* L. |  | NC_007944 |
| *Helianthus annuus* L. |  | NC_007977 |
| *Heliosperma alpestre* (Jacq.) Griseb. | Frajman 29.VI.2002 UPS | EU308519 |
| *Lactuca sativa* L. |  | NC_007578 |
| *Lotus japonicus* (Regel) K. Larsen |  | NC_002694 |
| *Lychnis abyssinica* (Hochst.) Lidén | Hedberg 5530 UPS | EU308531 |
| *Lychnis chalcedonica* L. Lc1 | Erixon 68 UPS | EU308503 |
| *Lychnis chalcedonica* L. Lc2 | -"- | EU308520 |
| *Lychnis chalcedonica* L. Lc3 | -"- | EU308521 |
| *Lychnis chalcedonica* L. Lc4 | -"- | EU308522 |
| *Lychnis flos-cuculi* L. | Oxelman 2200 GB | EU308523 |
| *Lychnis flos-jovis* Desr. | Oxelman ITS-FLO 30610 GB | EU308524 |
| *Lycopersicon esculentum* Mill. |  | NC_007898 |
| *Medicago truncatula* Gaertn. |  | AC093544 |
| *Nicotiana tabacum* L. |  | NC_001879 |
| *Nuphar advena* (Aiton) W.T. Aiton |  | DQ069357 |
| *Nymphaea alba* L. |  | NC_006050 |
| *Oenothera elata ssp. hookeri* (Torr. & A. Gray) W. Dietr. & W. L. Wagner | Erixon 104 UPS | EU308525 |
| *Oenothera elata ssp. hookeri* |  | NC_002693 |
| *Oenothera flava* Garrett | Erixon 102 UPS | EU308532 |
| *Oenothera fruticosa* L. | Erixon 101 UPS | EU308526 |
| *Oenothera* *macrocarpa (=missouriensis)* Pursh | Erixon 103 UPS | EU308527 |
| *Oryza sativa* L. clpP1 |  | NC_001320 |
| *Oryza sativa* L. clpP2 |  | XM_473235 |
| *Oryza sativa* L. clpP3 |  | AP003217 |
| *Oryza sativa* L. clpP4 |  | NM_197930 |
| *Oryza sativa* L. clpP5 |  | AB116073 |
| *Oryza sativa* L. clpP6 |  | AC092262 |
| *Panax ginseng* C.A. Mey. |  | NC_006290 |
| *Phalaenopsis aphrodite* Rchb. f. |  | NC_007499 |
| *Pinus thunbergii* Parl. |  | NC_001631 |
| *Ranunculus macranthus* Scheele |  | DQ069358 |
| *Saccharum officinarum* L. |  | NC_006084 |
| *Silene aegyptiaca* (L.) L.f. | Edmondson & McClintock 2933 E | EU308517 |
| *Silene atocioides* Boiss. | Oxelman 1690 GB | EU308518 |
| *Silene conica* L. | Erixon 70 UPS | EU308510 |
| *Silene conoidea* L. | Rautenberg 290 UPS | EU308528 |
| *Silene cryptoneura* Stapf | Oxelman 1628 GB | EU308512 |
| *Silene fruticosa* L. Sf1 | Oxelman & Tollsten 934 GB | EU308514 |
| *Silene fruticosa* L. Sf2 | -"- | EU308529 |
| *Silene fruticosa* L. Sf3 | -"- | EU308530 |
| *Silene integripetala* Bory & Chaub. | Oxelman 1902 GB | EU308509 |
| *Silene latifolia* Poir. | Erixon 72 UPS | EU308511 |
| *Silene littorea* Brot. | Oxelman 11.IV.1986 GB | EU308505 |
| *Silene pseudoatocion* Desf. | Erixon 71, UPS | EU308515 |
| *Silene samia* Melzh. & D. Christodoulakis | Oxelman 2208 UPS | EU308504 |
| *Silene schafta* S.G.Gmel. ex Hohen. | Popp 1053 UPS | EU308516 |
| *Silene sordida* Hub.-Mor. & Reese | Oxelman 2206 GB | EU308513 |
| *Silene sorensenis* (B.Boivin) Bocquet | Eggens 48 UPS | EU308508 |
| *Silene uniflora* Roth | Erixon 73 UPS | EU308506 |
| *Silene zawadskii* Hort. ex Fenzl | Oxelman 2241 GB | EU308507 |
| *Solanum bulbocastanum* Dunal |  | NC_007943 |
| *Spinacia oleracea* L. |  | NC_002202 |
| *Triticum aestivum* L. |  | NC_002762 |
| *Typha latifolia* L. |  | DQ069359 |
| *Vitis vinifera* L. |  | NC_007957 |
| *Yucca schidigera* Roezl ex Ortgies |  | DQ069360 |
| *Zea mays* L. |  | NC_001666 |
