# Supplementary material for: Whole-Gene Positive Selection, Elevated Synonymous Substitution Rates, Duplication, and Indel Evolution of the Chloroplast clpP1 Gene
Source: PLoS One. 2008 Jan 2;3(1):e1386. doi: 10.1371/journal.pone.0001386 (PMC2148103; doi:10.1371/journal.pone.0001386)
Supplement: Table S2 — Specific primers (0.02 MB DOC) [file pone.0001386.s003.doc]

Table S2. Specific primers

| Primer name | Sequence (5'-3') | Application |
| --- | --- | --- |
| ^a^clpP-F | TGAGCTTGGGCTTCTGTTGCTGACAT | *O. elata* |
| Oe-rps12spacer-F | GTTTCTTCCCGGATGCGTTGAC | *O. flava*/*fruticosa*/*macrocarpa* |
| Oe-clpP-R | CAATGGCATCCTTTCTACTGG | *O. macrocarpa* |
| Ofruti-e2-R | GTTGGGCTGATGGTATWTCTC | *O. fruticosa* |
| Oflava-i1-R1 | TCTCCCCCGATCGAGATATC | *O. flava* |
| Oflava-i1-R2 | AAGGACCCAGGCTCTGTTTAG | *O. flava* |
| Oflava-e2-R | GGATTAGSYATTTMTGATACTATG | *O. flava* |
| Oflava-i2-F | AACAAATGGTTCCTCTATCTCG | *O. flava* |
| clpP/psbH-F2b | GATAGATCATACTTGATAATGTCAAC | *L. chalcedonica*_Lc1 |
| clpP-Fspecial | CCCCTCTAAGATCAACATTCC | *L. chalcedonica*_Lc3 |
| clpPi2NY-F | CCTTCGATACATAATCTAGTGAG | *L. chalcedonica*_Lc3 |
| clpPi2F-frut | AGGTTTGTGACACKGAAACG | *S. fruticosa*_Sf1/Sf2 |
| Sf2-F | AGATAAATTCTCCCGTTTTAGCC | *S. fruticosa*_Sf2 |
| Sf2-R | TAAGGGGTTAGCTGCTTCAACGG | *S. fruticosa*_Sf2 |
| clpP/psbH-R6c | CTACTTCTACTACTACTACGACCCC | *S. fruticosa*_Sf1 |

^a^Universal primer used in [18].
